# The Evolving Interplay Between Targeted Therapy and Surgery for Resectable Lung Cancer

**DOI:** 10.3390/cancers17213575

**Published:** 2025-11-05

**Authors:** Victoria Yin, Mara B. Antonoff

**Affiliations:** 1 Michael E. DeBakey Department of Surgery, Baylor College of Medicine, Houston, TX 77030, USA; victoria.yin@bcm.edu; 2 Division of Thoracic and Cardiovascular Surgery, Department of Surgery, MD Anderson Cancer Center, Houston, TX 77030, USA

**Keywords:** targeted therapy, non-small cell lung cancer, surgery, tyrosine kinase inhibitor, osimertinib, alectinib, molecular testing, adjuvant therapy, neoadjuvant therapy

## Abstract

**Simple Summary:**

Lung cancer is one of the most common and deadly cancers worldwide. Surgery has been the main treatment for patients whose tumors can be removed, but many patients still experience recurrence after surgery. Novel medicines called targeted therapies can greatly improve outcomes for patients whose tumors have specific genetic changes. In this narrative review, we discuss landmark clinical trials of targeted therapies in resectable non-small cell lung cancer. We highlight how these therapies, when given before or after surgery, can lower the chances of cancer returning or improve survival. We also emphasize the importance of genetic testing to guide treatment planning. By bringing together the latest research, this review shows how targeted therapies are changing the treatment of resectable non-small cell lung cancer.

**Abstract:**

**Background**: Recent landmark clinical trials have introduced the role of targeted therapy with surgery for resectable non-small cell lung cancers (NSCLCs). **Methods**: This narrative review summarizes data from recent clinical trials and retrospective studies to highlight the evolving interplay between targeted therapy and resectable NSCLC. **Results**: For patients with epidermal growth factor receptor (*EGFR*) mutations, the ADAURA trial demonstrated significant improvements in disease-free and overall survival with adjuvant osimertinib after complete resection. The NeoADAURA trial expanded the role of osimertinib to neoadjuvant treatment as it showed benefit in major pathologic response rates when compared to chemotherapy alone. Neoadjuvant osimertinib may facilitate surgical resection, especially for patients with lymph node involvement. Furthermore, the ALINA trial established the role of adjuvant alectinib, another targeted therapy, for patients with anaplastic lymphoma kinase (*ALK*) positive resectable NSCLC. Given the evidence for use of these novel targeted therapies in patients with resectable lung cancer, early molecular profiling is critical for patients with NSCLC to help guide pre- and postoperative treatment. The use of targeted therapies may even expand to stage IV NSCLC as clinical trials are ongoing and could possibly redefine the role of surgery in advanced disease. **Conclusions**: While there are ongoing trials to clarify the optimal timing of targeted therapies and surgical resection, current data supports the use of targeted therapies as part of multimodality care in surgically resectable NSCLC.

## 1. Background

Lung cancer is the leading cause of cancer-related deaths worldwide, with over 2 million new cases and 1.8 million deaths annually [1]. The predominant histological subtype of lung cancer, non-small-cell lung cancer (NSCLC), has been historically managed according to disease extent as the key factor in treatment strategy. For the early stage of localized resectable NSCLC, surgery to remove the tumor is often first line. If the tumor size is large or has nodal involvement, then systemic therapy comes into play either in the neoadjuvant, adjuvant, or perioperative setting [2]. Chemotherapy has been associated with modest improvements to overall survival and disease-free survival; however, recurrence rates remain quite high. One-third to one-half of patients may recur after curative resection [3].

**Figure 1:**
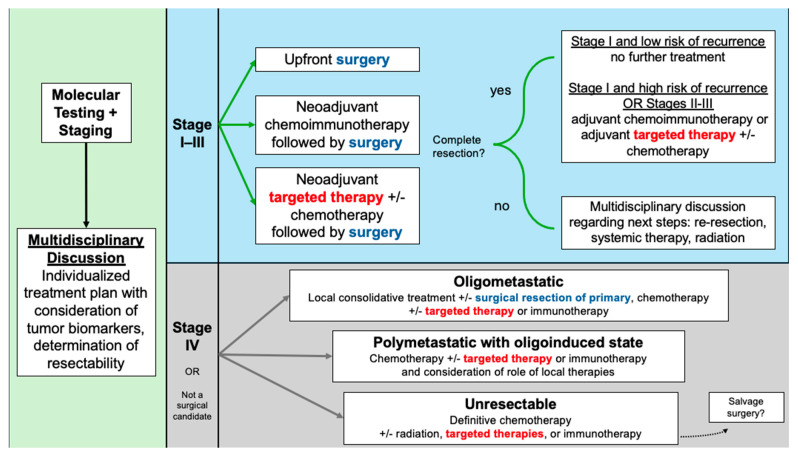
Treatment algorithm for resectable NSCLC and considerations in advanced NSCLC.

While the results of staging studies have been the cornerstone of determining individualized NSCLC care plans, newer paradigms have emphasized the importance of incorporating molecular profiling into the decision-making process (Figure 1). Lung cancer prognoses have improved significantly with the use of tyrosine kinase inhibitors (TKIs) for patients with sensitizing mutations. Most commonly, these are epidermal growth factor receptor (*EGFR*) alterations, which occur in 10–15% of Caucasian patients and approximately 50% of Asian patients with NSCLC [4]. Another clinically relevant alteration in NSCLC involves anaplastic lymphoma kinase (*ALK*), an oncogenic tyrosine kinase, with *ALK* rearrangements occurring in 3–7% of cases [4]. *ALK*-rearranged NSCLC, like *EGFR*-mutated, tends to be associated with younger patients, diagnosis at more advanced stages, and greater risk of brain metastasis [5]. In addition to *EGFR* and *ALK*, a spectrum of less common oncogenic drivers—including *ROS1*, *BRAF*, *MET*, *RET*, *NTRK*, and others—has been identified, with targeted therapies either available or under active investigation, highlighting the growing complexity and precision of NSCLC management [5,6].

## 2. Targeted Therapies for *EGFR* Mutations

### 2.1. ADAURA Trial

Targeted therapies were proven to be effective in patients with advanced stage NSCLC in clinical trials. The first generation TKI erlotinib was superior to chemotherapy in patients with stage IIIB or IV NSCLC harboring *EGFR* mutations [7,8]. This paved the groundwork for further investigations of erlotinib and gefitinib for *EGFR* mutations in stage III NSCLC. The EMERGING-CTONG 1103 trial was one of the first phase II randomized clinical trials of neoadjuvant and adjuvant erlotinib versus gemcitabine and cisplatin in resectable stage IIIA-N2 *EGFR*-mutated NSCLC and showed that erlotinib may improve progression-free survival and overall survival [9]. These studies demonstrated potential benefits in survival and responsiveness towards TKIs [7,8,9,10,11]. A third-generation TKI, osimertinib, was first approved by the U.S. Food and Drug Administration (FDA) in 2015, under an accelerated approval pathway for the treatment of patients with metastatic NSCLC harboring the *EGFR* T790M mutation, following progression on earlier generations of TKI [5,12]. The FLAURA trial demonstrated that osimertinib was associated with improved progression-free survival (PFS) and overall survival (OS) over gefitinib or erlotinib [13].

With promising data for osimertinib, investigators performed a double-blind, randomized controlled trial to explore the efficacy of the drug in resectable disease (ADAURA) [14]. This study population included patients with stage IB through IIIA NSLSC and Ex19del or L858R *EGFR* mutations. Randomization was assigned after surgery and chemotherapy in a 1:1 ratio according to stage, race, and mutation type. Patients received either 80 mg of oral osimertinib daily (N = 339) or placebo (N = 343) for three years. The primary outcome was disease-free survival (DFS) for patients with stage II to IIIA disease. At 24 months, DFS in the osimertinib group was 90% and 44% in the placebo group, indicating a significant reduction. In the overall population, DFS at 24 months was 89% in the osimertinib group and 52% in the placebo group. Amongst all subgroups (by sex, age, smoking history, race, stage, type of *EGFR* mutation, and adjuvant chemotherapy), osimertinib outperformed placebo in DFS. CNS-related recurrence was significantly reduced in the osimertinib group (2%) versus the placebo group (11%). Importantly, adverse events of grade 3 or higher were noted in 20% of osimertinib patients and 13% of placebo, indicating relative safety of osimertinib.

These encouraging results of osimertinib demonstrate the need for upfront genetic testing in NSCLC, especially for those who have had resected NSCLC. The ADAURA investigators later published results of five-year follow up data. They found the five-year overall survival in the osimertinib group to be 88% versus 78% in the placebo group, which was a statistically significant difference [15]. There was only one new serious adverse event with the additional data, which demonstrates the relative safety and tolerability of osimertinib that was shown in the original study [15]. While the ADAURA trial examined adjuvant osimertinib for three years, the ongoing TARGET trial is looking at a single arm of osimertinib for five years among patients with resected stage II to IIIB NSCLC to determine the optimal duration of adjuvant osimertinib [16]. Another ongoing trial similar to the ADAURA trial is ADAURA2, which examines patients with stage IA2 or IA3 *EGFR*-positive NSCLC on osimertinib versus placebo for 3 years [17]. Positive results from this trial would expand the role of adjuvant osimertinib after surgical resection in earlier stages of NSCLC than the ADAURA trial.

### 2.2. NeoADAURA Trial

Recently, the initial results of the first randomized trial with osimertinib in the neoadjuvant setting were released (NeoADAURA) [18]. Investigators enrolled patients with stage II to IIIB NSCLC with either Ex19del or L858R *EGFR* mutations who were deemed completely resectable by a multidisciplinary team. Patients were randomly assigned in a 1:1:1 ratio for ≥9 weeks of neoadjuvant therapy: 80 mg of oral osimertinib daily and platinum-based chemotherapy (N = 121); 80 mg of oral osimertinib daily monotherapy (N = 117); placebo- and platinum-based chemotherapy (N = 120). Randomization was stratified by disease stage, race, and *EGFR* mutation type. The primary outcome was major pathologic response (MPR). Following their neoadjuvant regimen, 98% patients went on to complete surgery, while the remaining patients did not due to progressive disease. There were no increases in postoperative complication rates with neoadjuvant osimertinib, demonstrating that neoadjuvant use does not impact perioperative outcomes and resectibility when compared to neoadjuvant chemotherapy. Patients who had neoadjuvant osimertinib and chemotherapy or osimertinib monotherapy had significantly higher rates of MPR than patients with placebo and neodjuvant chemotherapy, and this result was consistent among all subgroups. In addition, the rate of nodal downstaging at the time of surgery was higher for patients with osimertinib plus chemotherapy (53%) and osimertinib monotherapy (53%) compared to placebo and chemotherapy (21%). However, rates of pathological complete response (pCR) were quite low across all groups, with 4% for osimertinib plus chemotherapy, 9% for osimertinib monotherapy, and 0% for placebo plus chemotherapy, which may limit clinical applications and influence the results of longer-term overall survival which have not been published yet. It is important to note that this trial was not designed to determine whether neoadjuvant osimertinib would cause a previously borderline resectable tumor to be resectable, as all patients enrolled had resectable disease as a requirement [18]. As downstaging has been proposed to be the benefit of neoadjuvant therapies, further studies are necessary to determine if osimertinib could aid in resectability [19].

Multidisciplinary decision-making is essential when considering the optimal timing of surgery and neoadjuvant targeted therapy. While the NeoADAURA trial performed surgery after completion of at least 9 weeks of neoadjuvant osimertinib, further studies are necessary to determine the optimal duration and timing of neoadjuvant osimertinib with surgical resection [18]. Upfront molecular testing for sensitizing mutations should influence treatment planning as the *NeoADAURA* trial demonstrated superior results in patients who were taking osimertinib. In the absence of sensitizing mutations, the current standard of care is neoadjuvant chemotherapy in resectable NSCLC. However, chemotherapy is associated with higher rates of hematologic toxicities and adverse events when compared to osimertinib. Thus, it is crucial to offer patients osimertinib if they harbor sensitizing mutations as it has been shown to be better tolerated and have increased major pathologic response rates [18]. Standardization and reflexive testing protocols for molecular testing may need to be implemented nationwide as a recent study of the Medicare Surveillance, Epidemiology and End Results database found that there was substantial incongruence between patients receiving molecular testing and those receiving targeted therapy [20]. The authors found that only about half of the patients who were receiving targeted therapies had undergone prior molecular testing [20].

## 3. Targeted Therapies for *ALK* Mutations

### ALINA Trial

Alectinib, an *ALK*-TKI, was first used in the phase 3 ALEX study for advanced stage *ALK*-positive NSCLC [21]. Its efficacy appeared promising against a first-generation *ALK*-TKI and was therefore investigated further under the ALINA trial. The ALINA trial is a phase 3 randomized trial which enrolled patients with completely resected stage IB, II, or IIIA NSCLC with *ALK*-rearranged disease [22]. Patients were randomly assigned to 600 mg of oral alectinib twice daily for 24 months (N = 130) or intravenous platinum-based chemotherapy for four 21-day cycles (N = 127) in a 1:1 ratio, stratified by disease stage and race. The primary outcome was DFS. At 2 years, DFS among patients taking alectinib was 93.6% versus 63.7% in the chemotherapy group. This differential remained profound at 3 years, with 88.7% DFS in the alectinib group versus 54.0% in the chemotherapy group. This was also consistent among all subgroups studied: age, race, sex, Eastern Cooperative Oncology Group (ECOG) performance status, disease stage, regional lymph node status, and smoking status [22].

The ALINA trial resulted in the approval of alectinib by the U.S. Food and Drug Administration (FDA) as an adjuvant therapy for patients with *ALK*-rearranged NSCLC [5]. Further studies are necessary to investigate the ideal treatment length of adjuvant alectinib. In addition, the ALNEO trial is currently investigating the role of alectinib as neoadjuvant therapy for patients with stage III resectable NSCLC [23]. A summary of key clinical trials with targeted therapies for *EGFR* and *ALK* can be found in Table 1.

## 4. Targeted Therapies for Other Mutations

In addition to *EGFR* and *ALK* alterations, a spectrum of less common oncogenic drivers has been identified in NSCLC, including *ROS1*, *BRAF* V600E, *MET* exon 14 skipping, *RET*, *NTRK*, *KRAS* G12C, and *HER2* mutations (Table 2) [24]. *ROS1* rearrangements, present in approximately 1–2% of cases, are sensitive to crizotinib and entrectinib in advanced disease, though data in the adjuvant or neoadjuvant setting remain limited [25]. *BRAF* V600E mutations occur in 1–3% of NSCLC, and targeted therapy with dabrafenib plus trametinib demonstrates efficacy in metastatic disease, with early-phase trials now exploring their role after resection [26,27]. *MET* exon 14 skipping mutations, found in 3–4% of patients, and *RET* fusions, present in 1–2%, are both actionable with *MET* inhibitors (capmatinib, tepotinib) and *RET* inhibitors (selpercatinib, pralsetinib), though adjuvant investigations are still underway [28,29]. *NTRK* fusions are rare (<1%) but highly responsive to pan-TRK inhibitors such as larotrectinib and entrectinib, and ongoing basket trials continue to explore these agents in earlier-stage disease [5,30]. Emerging targets such as *KRAS* G12C (~13%) and *HER2* (~2–3%) also have FDA-approved therapies in metastatic NSCLC, with early-phase studies evaluating their incorporation into resectable disease management [5,31]. Collectively, these developments underscore the rapidly evolving landscape of precision-targeted therapy in NSCLC, and ongoing and planned clinical trials will help define the role of specific agents for these less common mutations in both the adjuvant and neoadjuvant settings.

For patients without oncogenic driver mutations, immune checkpoint inhibitors (ICIs) have been changing the perioperative treatment of resectable NSCLC. These agents are antibodies against programmed death 1 (PD-1) or programmed death-ligand 1 (PD-L1) and include nicolumab, pembrolizumab, atezolizumab, and durvalamab, which all currently have FDA approval in NSCLC. The CheckMate816 trial examined neoadjuvant nivolumab in patients with resectable stage IB–IIIA NSCLC without *EGFR* or *ALK* mutations and found that neoadjuvant nivolumab with chemotherapy resulted in improved pathologic complete response and event-free survival compared to chemotherapy alone [32]. More recent clinical trials have evaluated preoperative and postoperative chemoimmunotherapy regimens including the AEGEAN trial (durvalamab), the NEOTORCH trial (toripalimab), and the KEYNOTE-671 trial (pembrolizumab) which have consistently demonstrated improvement in event-free survival and pathologic complete response compared to conventional chemotherapy [33,34,35]. It is important to note that the efficacy of immunotherapy was most profound in patients with high PD-L1 expression or advanced disease. There are ongoing efforts to better elucidate patient selection and optimal neoadjuvant plus/minus adjuvant chemoimmunotherapy regimens [36].

## 5. Early Molecular Testing

The striking evidence for the use of targeted therapies suggests that patients with NSCLC should be routinely screened for certain mutations. There is a need for rapid testing for targetable mutations (*EGFR*, *ALK*, *ROS1*, *BRAF*, *MET*, *RET*, etc.) across all stages of NSCLC [37]. Previously, genetic testing for *ALK* was routine for advanced NSCLC only. However, given the benefit of targeted therapies in resectable NSCLC that have been highlighted by the ADAURA, NeoADAURA, and ALINA trials, the pool of patients undergoing genetic testing should be expanded [22]. The current Society of Thoracic Surgeons statement recommends patients with resectable, locally advanced NSCLC with targetable driver mutations to undergo induction chemotherapy, followed by resection, and adjuvant targeted therapy plus/minus chemotherapy [38]. Therefore, with guidelines shifting towards the use of targeted therapy for all resectable NSCLC, genetic testing is a crucial aspect of treatment planning.

In cases of resectable NSCLC, the molecular testing for genetic mutations can be performed on the specimen; however, the method of detection for each mutation varies based off mechanism of alteration and expression of the mutation. These tests range from mutation-specific polymerase chain reaction (PCR) assays to immunohistochemistry and fluorescence in situ hybridization (FISH) [39]. This makes molecular testing logistically complicated and often times unfeasible if a patient has advanced NSCLC and has undergone biopsies with limited tissue yield. The emergence of next generation sequencing (NGS) can help solve this problem as it can detect multiple targets simultaneously, potentially improving efficiency for detecting a wide range of mutations [40]. Studies have shown similar sensitivity between NGS and conventional technologies with advantages in detecting variants with low mutation rates or detecting non-hotspot mutations [41,42]. However, NGS requires a greater upfront cost for expensive and modern equipment which may not be accessible for all institutions. It is typically performed in centralized laboratories which may actually result in longer turnaround times than conventional methods which can be performed locally [39]. Other logistical or operational barriers to rapid molecular testing include tissue processing, testing algorithms, and choice of assay (i.e., single gene versus panel-based) [43]. Delays in biomarker test results may potentially lead to physicians choosing inferior therapy in cases where there is clinical urgency to begin treatment [43].

Obtaining adequate tissue samples can be difficult, especially when there is not a surgical specimen as is the case in advanced or unresectable NSCLC or in testing for neoadjuvant targeted therapy. This is because there may not be sufficient specimen to run the multitude of tests if NGS is not available; a single biopsy molecular profile may not reflect the heterogeneity of the tumor, and patients may not be able to tolerate an invasive procedure. Plasma-based NGS testing on circulating tumor DNA (ctDNA) or “liquid biopsies” have been developed and are currently being assessed to determine if they can address the limitations with tissue-based testing [40]. Some studies suggest that plasma-NGS may be able to detect clinically relevant mutations at similar rates when compared to tissue-NGS [44,45]. As these novel methods continue to advance, liquid biopsy may become an important complement or alternative to tissue-based testing, helping ensure timely and accurate detection of targetable mutations.

## 6. Advanced Disease and Targeted Therapies

Targeted therapies have created opportunities for surgical intervention in selected advanced stage NSCLC patients. Due to their high response rates in tumors with sensitizing mutations, targeted therapies have the potential to increase the pool of patients who could benefit from surgical resection [46]. Retrospective studies of patients with advanced NSCLC who underwent definitive systemic treatment with *EGFR* TKI followed by salvage surgery were able to be down-staged in approximately one-quarter of cases prior to surgery [47]. While salvage surgery did not improve recurrence rates, it did result in improved three-year overall survival rates at approximately 75% likely due to better local control of TKI-resistant tumor regions [47,48]. These findings suggest that targeted therapies may allow selected patients with advanced NSCLC to have surgery included in their multimodality treatment plans.

Another advanced stage of NSCLC in which surgical intervention may be indicated is oligometastatic disease, which refers to tumors with limited metastatic spread that could undergo aggressive local treatment with curative intent (however, there lacks a consensus definition) [46,49,50]. In a retrospective study of patients with oligometastatic NSCLC who underwent surgical resection for the primary lung tumor and either complete or partial treatment for metastatic sites, patients who had adjuvant TKI therapy had superior survival compared to the remainder of the adjuvant chemotherapy or no treatment cohorts [51]. This highlights how surgery can fit into multimodality treatment regimens, especially in patients with oligometastatic disease harboring TKI-sensitizing tumor biology. There are several ongoing clinical trials to determine if targeted therapy in combination with local consolidative therapy improves survival outcomes in patients with stage IV NSCLC, including oligometastatic and polymetastatic disease, when compared to targeted therapy alone [49,52]. The highly anticipated NORTHSTAR trial (NCT03410043) compared osimertinib with or without local consolidative therapy (surgery and/or radiation) in stage IV NSCLC, with stratification by extent of disease (i.e., oligometastatic, polymetastatic), and results are anticipated in late 2025 [53,54]. Similarly, the BRIGHTSTAR trial (NCT03707938) is investigating the role of brigatinib with local consolidative therapy in patients who have *ALK*-rearranged advanced oligometastatic or polymetastatic NSCLC, with promising early results presented at the 2020 American Society of Clinical Oncology Annual Meeting [55,56]. Together, these studies may help define the role of surgery within multimodality treatment strategies for stage IV NSCLC and clarify how best to integrate targeted therapy with local consolidative approaches.

## 7. Conclusions

Further clinical trials are necessary to clarify the optimal timing of targeted therapies and surgical resection. At present, results from recent landmark clinical trials support the use of targeted therapies with surgically resectable NSCLC in neoadjuvant and adjuvant settings. Therefore, routine and rapid genetic testing among early-stage NSCLC is necessary to help dictate optimal treatment plans with targeted therapies and surgical resection.

## Figures and Tables

**Table 1:** Key clinical trials of *EGFR*- and *ALK*-targeted therapies in resectable NSCLC ^1^.

Trial	Mutation	Stages	Treatment Arm(s)	Endpoints	Key Results
ADAURA [13,14]	*EGFR* L858R or Ex19del	IB–IIIA	Adjuvant osimertinib versus placebo for 3 years	Primary: disease-free survival for stage II–IIIA disease Secondary: disease-free survival for stage I-IIIA disease, overall survival, safety	**Median DFS (stage II–IIIA)** Osimertinib not reached, 95% CI: 38.8-not calculable Placebo 19.6 mo, 95% CI 16.6–24.5 Hazard ratio for disease recurrence or death: 0.17, 95% CI 0.11–0.26, *p* < 0.001 **Five-year OS (Stage II–IIIA)** Osimertinib 85%, 95% CI 79–89% Placebo 73%, 95% CI 66–78% Hazard ratio for death: 0.49, 95% CI 0.33–0.73, *p* < 0.001
NeoADAURA [17]	*EGFR* L858R or Ex19del	II–IIIB	Neoadjuvant osimertinib plus chemotherapy versus osimertinib monotherapy versus placebo plus chemotherapy	Primary: major pathologic response Secondary: event-free survival	**Major pathologic response rate** Osimertinib plus chemotherapy 26% Osimertinib monotherapy 25% Placebo plus chemotherapy 2% Odds ratio for osimertinib plus chemotherapy versus placebo plus chemotherapy: 19.82, 95% CI 4.60–85.33, *p* < 0.0001 Odds ratio for osimertinib monotherapy versus placebo plus chemotherapy: 19.28, 95% CI 1.71–217.39, *p* < 0.0001 **Median EFS** Osimertinib plus chemotherapy not reached, 95% CI not calculable Osimertinib monotherapy not reached, 95% CI 30.3 months—not calculable Placebo plus chemotherapy not reached, 95% CI not calculable
TARGET [15]	*EGFR* Common mutations cohort: Ex19del or L858R Uncommon mutations cohort: G719X, L861Q, or S768I	II–IIIB	Adjuvant osimertinib for 5 years	Primary: disease-free survival at 5 years among patients with common *EGFR* mutations Secondary: disease free-survival at 3, 4 years (common mutations), overall survival at 3, 4, and 5 years, disease-free survival at 3, 4, and 5 years among patients with uncommon *EGFR* mutations, safety and tolerability, type of recurrence and CNS metastases	Ongoing, results expected in 2029
ADAURA2 [16]	*EGFR* L858R or Ex19del	IA2–IA3	Adjuvant osimertinib versus placebo for 3 years	Primary: disease-free survival among patients with high pathologic risk for disease recurrence Secondary: disease-free survival overall, overall survival, CNS disease-free survival, safety	Ongoing, results expected in 2027
ALINA [21]	*ALK* rearrangement	IB–IIIA	Adjuvant alectinib for 24 months versus chemotherapy for four 21-day cycles	Primary: disease-free survival (II-IIIA and intention-to-treat) Secondary: CNS disease-free survival, overall survival, safety	**Median DFS (II-IIIA)** Alectinib not reached, 95% CI not calculable Chemotherapy 44.4 months, 95% CI 27.8-not calculable Hazard ratio for disease recurrence or death: 0.24, 95% CI 0.13–0.45, *p* < 0.001 **Median DFS (intention-to-treat)** Alectinib not reached, 95% CI not calculable Chemotherapy 41.3 months, 95% CI 28.5-not calculable Hazard ratio for disease recurrence or death: 0.24, 95% CI 0.13–0.43, *p* < 0.001

^1^ DFS: disease-free survival; OS: overall survival; CI: confidence interval; EFS: event-free survival; CNS: central nervous system.

**Table 2:** Other actionable mutations in NSCLC: prevalence, targeted therapies, and ongoing trials.

Mutation	Prevalence	Targeted Therapies	Ongoing Trials
*ROS1*	1–2%	Crizotinib, entrectinib, iruplinalkib	NCT04084717: Phase 2, crizotinib in patients with metastatic or stage IV NSCLC with *ROS1* rearrangement. Primary endpoints: response rate, progression-free survival, average time-to-treatment failure, Edmonton Symptom Assessment Scale Score, EQ5D-DL Questionnaire Score, overall survival Neo-INFINITY (NCT05765877): Phase 2, neoadjuvant iruplinalkib in patients with resectable NSCLC containing *ALK* or *ROS1* mutations. Primary endpoint: major pathologic response rate NAUTIKA-1 (NCT04302025): Phase 2, neoadjuvant entrectinib in patients with resectable NSCLC containing *ROS1* fusion. Primary endpoint: major pathologic response
*BRAF* V600E	1–3%	Dabrafenib plus trametinib, vemurafenib plus cobimetinib	NCT06054191: Phase 2, patients with stage IB–IIIA NSCLC harboring *BRAF* V600E mutation will receive neoadjuvant and adjuvant dabrafenib plus trametinib. Primary endpoint: pathologic complete response rate NAUTIKA-1 (NCT04302025): Phase 2, neoadjuvant vemurafenib plus cobimetinib in patients with resectable NSCLC containing *BRAF* V600E mutation. Primary endpoint: major pathologic response rate
*MET* exon 14	3–4%	Capmatinib, tepotinib	NCT06054191: Phase 2, patients with stage IB–IIIA NSCLC harboring *MET* exon 14 skipping mutation will receive neoadjuvant and adjuvant capmatinib with surgery. Primary endpoint: pathologic complete response rate
*RET*	1–2%	Selpercatinib, pralsetinib	LIBRETTO-001 (NCT03157128): Phase 2, patients with advanced solid tumors harboring *RET* gene alteration. Cohort 7 includes early-stage NSCLC patients who are candidates for definitive surgery who will receive selpercatinib in neoadjuvant and adjuvant setting. Primary endpoint: objective response rate LIBRETTO-432 (NCT04819100): Phase 3, adjuvant selpercatinib following definitive locoregional treatment (surgery or radiation) versus placebo for patients with stage IB–IIIA *RET* fusion-positive NSCLC. Primary endpoint: event-free survival NAUTIKA-1 (NCT04302025): Phase 2, neoadjuvant pralsetinib in patients with resectable NSCLC containing *RET* fusion. Primary endpoint: major pathologic response rate
*NTRK*	<1%	Larotrectinib, entrectinib	NAVIGATE (NCT02576431): Phase 2, basket trial for larotrectinib which includes NSCLC patients with *NTRK*1/2/3 fusion. Primary endpoint: best overall response STARTRK-2 (NCT02568267): Phase 2, basket trial for entrectinib which includes patients with solid tumors which harbor *NTRK*1/2/3 rearrangement. Primary endpoint: objective response NAUTIKA-1 (NCT04302025): Phase 2, neoadjuvant entrectinib in patients with resectable *NTRK* mutant NSCLC. Primary endpoint: major pathologic response rate
*KRAS* G12C	13%	Sotorasib, divarasib	NCT05118854: Phase 2, neoadjuvant sotorasib plus chemotherapy in patients with surgically resectable *KRAS* G12C mutant NSCLC. Primary endpoints: major pathologic response rate in resected tumor specimens, safety, tolerability, and recommended phase 2 dose CodeBreaK 202 (NCT05920356): Phase 3, sotorasib plus chemotherapy versus pembrolizumab plus chemotherapy for patients with stage IIIB–IV *KRAS* G12C mutant, PD-L1 negative NSCLC. Primary endpoints: progression-free survival and overall survival NAUTIKA-1 (NCT04302025): Phase 2, divarasib as neoadjuvant treatment in patients with resectable *KRAS* G12C mutant NSCLC. Primary endpoint: percentage of patients with three to five adverse events, percentage of patients without delays of surgery due to treatment-related adverse events
*HER2*	2–3%	Trastuzumab deruxtecan, zongertinib	DESTINY-Lung04 (NCT05048797): Phase 3, Trastuzumab deruxtecan versus chemotherapy as first-line treatment of unresectable, locally advanced, or metastatic NSCLC harboring *HER2* exon 19 or 20 mutations. Primary endpoint: progression-free survival Beamion LUNG-1 (NCT04886804): Phase 1a-1b, zongertinib patients with advanced or metastatic *HER2*-mutant NSCLC who were previously treated. Primary endpoint: objective response

## Abbreviations

The following abbreviations are used in this manuscript:

NSCLC	Non-small cell lung cancer
EGFR	Epidermal growth factor receptor
TKI	Tyrosine kinase inhibitor
ALK	Anaplastic lymphoma kinase
PD-1	Programmed death 1
PD-L1	Programmed death-ligand 1
FDA	Food and Drug Administration
PFS	Progression-free survival
OS	Overall survival
DFS	Disease-free survival
MPR	Major pathologic response
ECOG	Eastern Cooperative Oncology Group
PCR	Polymerase chain reaction
NGS	Next generation sequencing
FISH	Fluorescence in situ hybridization
ctDNA	Circulating tumor DNA
CI	Confidence interval
EFS	Event-free survival
CNS	Central nervous system
